# Familial Consent for Deceased Organ Donation Among Immigrants and Long-term Residents in Ontario, Canada: A Population-Based Retrospective Cohort Study

**DOI:** 10.1177/2054358117735564

**Published:** 2017-10-20

**Authors:** Alvin Ho-ting Li, Ahmed A. Al-Jaishi, Matthew Weir, Ngan N. Lam, Janet Maclean, Sonny Dhanani, S. Joseph Kim, Greg Knoll, Amit X. Garg

**Affiliations:** 1 Department of Epidemiology and Biostatistics, Western University, London, Ontario, Canada; 2 Ottawa Hospital Research Institute, Ontario, Canada; 3 Institute for Clinical Evaluative Sciences, Toronto, Ontario, Canada; 4 Division of Nephrology, Western University, London, Ontario, Canada; 5 Division of Nephrology, University of Alberta, Edmonton, Canada; 6 Trillium Gift of Life Network, Toronto, Ontario, Canada; 7 Division of Critical Care, Children’s Hospital of Eastern Ontario, Ottawa, Canada; 8 Division of Nephrology, University of Toronto, Ontario, Canada; 9 Institute of Health Policy, Management and Evaluation, University of Toronto, Ontario, Canada; 10 Division of Nephrology, Department of Medicine, Ottawa, Ontario, Canada

**Keywords:** organ donation, immigrants, epidemiology

## Abstract

**Background:**

Many families choose not to consent to organ donation at the time of their loved one’s death. In Ontario, Canada, whether these decisions vary by ethnicity remains unclear.

**Objective:**

To compare the proportion of families of immigrants who consented for deceased organ donation with families of long-term residents.

**Design:**

Population-based retrospective cohort study.

**Setting:**

Potential donors in Ontario, Canada, between November 2008 and March 2013.

**Methods:**

We used linked administrative databases to study the proportion of families who consented for deceased organ donation.

**Results:**

Overall, of the 2873 families of potential donors approached, 1912 (67%) provided consent for deceased organ donation. Families of immigrants were less likely to provide consent compared with families of long-term residents (46% [135 of 291] vs 69% [1777 of 2582]; adjusted rate ratio (RR): 0.72; 95% confidence interval [CI]: 0.63-0.81). When examined by the country of birth, families of immigrants from the following regions were less likely to consent to organ donation compared with long-term residents: South Asia (RR: 0.71; 95% CI: 0.55-0.91), East Asia and Pacific (RR: 0.68; 95% CI: 0.53-0.88) and Middle East, North Africa, and sub-Saharan Africa (RR: 0.58; 95% CI: 0.37-0.91).

**Limitations:**

We could not determine why consent was not obtained. We had a small sample of immigrants. We only had access to the potential donors’ information and not the family member who was approached for consent. Many characteristics that we examined were nonmodifiable (eg, age, sex).

**Conclusions:**

In Ontario, families of immigrants are less likely to consent to deceased organ donation. There is an opportunity to better understand the reasons for lower consent among certain immigrant groups.

## Abrégé

**Contexte:**

À la mort d’un être cher, plusieurs familles refusent de consentir au don d’organes. Au Canada, particulièrement dans la province de l’Ontario, il demeure difficile d’établir si une telle décision varie selon l’origine ethnique des personnes concernées.

**Objectif de l’étude:**

L’étude visait à comparer la proportion de familles issues de l’immigration ayant consenti au don d’organe après le décès d’un proche à celle de familles résidant au pays depuis longtemps.

**Type d’étude:**

Il s’agit d’une étude de cohorte rétrospective basée sur une population.

**Cadre de l’étude:**

On a ciblé les familles résidant en Ontario, au Canada, qui avaient été abordées entre septembre 2008 et mars 2013 en vue de faire don des organes et des tissus d’un proche décédé.

**Méthodologie:**

Nous avons utilisé les bases de données couplées du système de santé pour établir la proportion de familles ayant consenti au don d’organes d’un proche décédé.

**Résultat:**

Dans l’ensemble, la majorité des 2 873 familles abordées, soit 1 912 (67 %), a consenti au don d’organes de leur proche décédé. Nous avons toutefois constaté que les familles issues de l’immigration se sont montrées plus réticentes à consentir au don d’organes que les familles de résidents de longue date (46 % [135 of 291] contre 69 % [1777 of 2582]; RR à 0,72 %; IC 95 % : 0,63 à 0,81). La comparaison sur la base du pays d’origine a permis d’établir que les personnes en provenance des régions suivantes étaient plus susceptibles de refuser de donner leur consentement : Asie du Sud (RR à 0,71; IC 95 % : 0,55 à 0,91), Asie de l’Est et du Pacifique (RR à 0,68; IC 95 % : 0,53 to 0,88), Moyen-Orient, Afrique du Nord et Afrique subsaharienne (RR à 0,58; IC 95 % : 0,37 to 0,91).

**Limites:**

La portée des résultats de cette étude pourrait être limitée par plusieurs facteurs : notamment, nous n’avons pas pu obtenir d’information sur les raisons du refus de consentement, nous n’avions accès qu’aux renseignements du donneur potentiel et non à ceux des membres de la famille abordés pour la demande de consentement. Ajoutons à cela la taille restreinte de l’échantillon des familles issues de l’immigration et le fait que plusieurs des caractéristiques examinées n’étaient pas modifiables (l’âge ou le sexe, par exemple).

**Conclusion:**

Cette étude conclut qu’en Ontario, les familles issues de l’immigration sont plus réticentes à consentir au don d’organes et de tissus d’un proche décédé. Dès lors, une occasion nous est offerte pour tenter de mieux comprendre les raisons qui expliquent cette faible proportion de consentement chez certains groupes d’immigrants.

## What was known before

Many families choose not to consent to organ donation at the time of their loved one’s death. Families of critically ill ethnic minorities may be less likely to consent on their relative’s behalf.

## What this adds

These results suggest that in Ontario, families of immigrants are less likely to consent to deceased organ donation. There is an opportunity to identify and better understand the reasons for lower rate consent among certain immigrant groups.

## Background

The ongoing shortage of organs for transplantation demands strategies that maximize the availability of this scarce resource. Several countries are working to increase the proportion of families who consent to deceased organ donation at the time of their relative’s death, which ranges between 40% and 70% across jurisdictions.^1-3^ Consent rates are undoubtedly a significant factor for increasing transplantation across all ethnicities. Ethnic minorities have been shown to have lower rates of organ donation registration, and families of critically ill ethnic minorities may be less likely to consent on their relative’s behalf.^2,4^ Therefore, this population may represent an important source of underutilized organs that could be better accessed through culturally sensitive education programs.

Canada has the highest proportion of foreign-born individuals among the 8 leading industrial and developed countries in the world, with the majority of immigrants living in its most populated province, Ontario.^5^ The province also houses some of the most comprehensive, large, administrative health care databases in the country, which facilitates population-level health research. Using these resources, we compared the familial consent rates of immigrants with long-term residents in general, and by region of origin. We also identified patients who were already registered donors to see whether registration modified the likelihood of obtaining final consent from families.

## Methods

### Study Design and Setting

We conducted a population-based retrospective cohort study in the province of Ontario, Canada (population: 13 million) using large administrative health care databases held at the Institute for Clinical Evaluative Sciences (ICES). These datasets were linked using unique encoded identifiers. In Ontario, residents have universal health care coverage. To be an organ donor, the decedent must have suffered a nonrecoverable injury and be mechanically ventilated at the time the provincial organ procurement organization is notified.^6^ Each decedent’s eligibility to donate is evaluated on an individual basis. At the time of imminent death or family’s interest in organ donation, a donor coordinator experienced in talking to families about donation will access the decedent’s donor registration information. If the decedent is registered, the coordinator will provide this information to the donor’s family members at an appropriate time. In Ontario, the next-of-kin makes the final decision on proceeding with organ and tissue donation regardless of whether the decedent, prior to death, was registered for organ and tissue donation or not. We did not include decedents who were only referred for tissue-only donation. We conducted this study according to a prespecified protocol that was approved by the research ethics boards at Sunnybrook Health Sciences Centre (Toronto, Canada). We used the RECORD statement to guide the reporting of this study (Supplemental Table 1).

### Data Sources

We ascertained demographic information, potential confounders, and outcome information of potential donors from linked administrative databases. The second author (A.A.A) had access to the database population used to create the study population.

First, we obtained data of those who were referred for deceased organ and tissue donation from the Trillium Gift of Life Network. Patients who meet any of the following are referred for potential organ donor consideration: (1) Glasgow Coma Scale score of 3, (2) injured brain or nonrecoverable injury or illness, (3) family initiated discussion of organ donation with the health care team or withdrawal of life-sustaining therapy, and/or (4) planned discussion of therapy limited, de-escalation of treatment, or withdrawal of life-sustaining therapy. All patients referred for organ donation are then evaluated for medical suitability. This dataset also contains information on whether the family was approached for donation and whether consent from their family was obtained. These data are recorded on a real-time basis by a call center. We refer to these patients as potential donors.

Second, we obtained demographics from the Ontario Registered Persons Database. This database has demographic and vital status information on all residents who have ever been issued a health card. We estimated the individual’s income using neighborhood income quintiles.

Third, we used Matheson’s Canadian Marginalization Index to assign marginalization quintiles.^7^ This index describes 4 components of marginalization: residential instability, ethnic concentration, dependency, and material deprivation.^7^

Fourth, we used the Canadian Institute for Health Information Discharge Abstract Database (CIHI-DAD) to obtain information on hospitalizations to ascertain the patient’s cause of death and to determine whether the admitting hospital of the potential donor had an academic affiliation. We classified cause of death using the International Classification of Diseases Revision 10 codes into traumatic brain injury (S06, S07, S08, S09); subarachnoid and intracranial hemorrhage (I60, I61, I62); other damage to the brain (I63, I64); acute myocardial infarction, cardiac arrest (I21, I22, I23, I46); and all other causes of death.

Finally, we used the Immigration, Refugees and Citizenship Canada’s (IRCC) Permanent Resident Database to ascertain immigration status. This database contains landing records for every permanent legal immigrant who landed in Canada since 1985 onward. All information is captured at the time of immigration application. The overall linkage rate of this dataset to the Registered Persons Database is approximately 86.4%.^8^ More details about the linkage and the dataset can be found elsewhere.^8^ We generally grouped each immigrant’s country of birth by their world region of origin, according to the World Bank system: (a) South Asia; (b) East Asia and Pacific; (c) Latin America and Caribbean; (d) the United States, Australia, New Zealand, and Western Europe; (e) Middle East, North Africa, and sub-Saharan Africa; and (f) Eastern Europe and Central Asia.^9^ We combined Western Europe with the United States, Australia, and New Zealand in one group, as well as sub-Saharan Africa, Middle East, and North Africa in another group because of small sample sizes. This grouping is done because we hypothesized that differences in familial consent are primarily due to cultural awareness and attitudes.

### Study Population, Outcomes, and Statistical Analysis

We included all permanent residents of Ontario who were approached for deceased organ and tissue donation with a record of hospitalization from November 1, 2008, to March 31, 2013. For our comparison of immigrants and long-term residents, we classified immigrants as having a record within the IRCC’s Permanent Resident Database. Everyone else without a record in the IRCC database was classified as long-term residents (including immigrants who landed in Canada prior to 1985).

The outcome of interest was obtaining consent from the families of potential donors. We assessed differences in baseline characteristics between immigrants and long-term residents using the chi-square test for categorical variables and the Mann-Whitney *U* test for continuous variables. We used a modified Poisson regression model with a robust error estimator to compare familial consent rates among immigrants and long-term residents. We used complete-case analysis as the amount of missing data was low (~2%). We also evaluated the association between immigrant status and familial consent in 4 subgroups: age, sex, hospital type, and cause of death. We determined *P* values for interaction by including the interaction terms in the regression models. We hypothesized that these 4 characteristics affect long-term residents and immigrants similarly. We adjusted for 11 potential confounders: world region of birth, age, sex, residence (urban vs rural), neighborhood income quintile, material deprivation, ethnic concentration, dependency, residential instability, cause of death, and academic hospital affiliation. We conducted analyses using SAS software version 9.4 (SAS Institute Inc, Cary, North Carolina), and a 2-sided *P* value <.05 was considered to be statistically significant.

## Results

From November 1, 2008, to March 31, 2013, there were 2926 potential donors approached to obtain familial consent (Supplemental Figure 1). Of the 2926 potential donors, 291 were immigrants and 2635 were long-term residents. The median age of immigrants was 54 (interquartile range [IQR]: 42-65) and median age of long-term residents was 57 (IQR: 44-68). The baseline characteristics for the potential donors whose families were approached for consent by immigration status are presented in Table 1. The groups differed on most baseline characteristics; immigrants were more likely to be younger, live in areas with lower income, and demonstrate higher levels of marginalization. Of the 2926 potential donors, no immigrants and 53 long-term residents (2%) had missing data on the marginalization quintiles. Thus, 2873 potential donors were used to determine the rate of familial consent among immigrants and long-term residents.

**Table 1.** Baseline Characteristics of Deceased Immigrants and Long-term Residents Whose Families Were Approached for Organ and Tissue Donation.

Characteristic	Immigrant (n = 291), n (%)	Long-term residents (n = 2635), n (%)	*P* value
Age			*0.016*
<18 y	8 (2.7)	153 (5.8)	
18 to <45 y	75 (25.8)	518 (19.7)	
45 to <65 y	134 (46.0)	1093 (41.5)	
≥65 y	74 (25.4)	871 (33.1)	
Women	120 (41.2)	1060 (40.2)	.7391
Rural residence^a^	≤5^b^	367 (14)	*<.0001*
Income quintile^c^			*<.0001*
First (lowest)	90 (30.9)	584 (22.2)	
Second	69 (23.7)	543 (20.6)	
Third (middle)	67 (23.0)	535 (20.3)	
Fourth	38 (13.1)	525 (19.9)	
Fifth (highest)	27 (9.3)	448 (17.0)	
Residential instability^d^			*<.0001*
First (lowest)	92 (31.6)	565 (21.4)	
Second	42 (14.4)	540 (20.5)	
Third (middle)	28 (9.6%)	400 (15.2)	
Fourth	72 (24.7)	499 (18.9)	
Fifth (highest)	57 (19.6)	578 (21.9)	
Missing	0	53 (2.0)	
Ethnic concentration^e^			*<.0001*
First (lowest)	≤5^b^	410 (15.6)	
Second	≤10^b^	444 (16.9)	
Third (middle)	23 (7.9)	509 (19.3)	
Fourth	31 (10.7)	570 (21.6)	
Fifth (highest)	225 (77.3)	649 (24.6)	
Missing	0	53 (2.0)	
Dependency^f^			*<.0001*
First (lowest)	89 (30.6)	457 (17.3)	
Second	76 (26.1)	576 (21.9)	
Third (middle)	70 (24.1)	532 (20.2)	
Fourth	37 (12.7)	484 (18.4)	
Fifth (highest)	19 (6.5)	533 (20.2)	
Missing	0	53 (2.0)	
Material deprivation^g^			.422
First (lowest)	51 (17.5)	554 (20.6%)	
Second	60 (20.6)	574 (21.8)	
Third (middle)	65 (22.3)	529 (20.1)	
Fourth	58 (19.9)	488 (18.5)	
Fifth (highest)	57 (19.6)	437 (16.6)	
Missing	0	53 (2.0)	
Hospital type where death occurred			*<.0001*
Academic hospital	126 (43.3)	1584 (60.1)	
Community hospital	165 (56.7)	1051 (39.9)	
Cause of death			*<.0004*
Traumatic brain injury	54 (18.6)	449 (17.0)	
Subarachnoid and intracranial hemorrhage	92 (31.6)	618 (23.5)	
Other damage to the brain	25 (8.6)	148 (5.6)	
Acute myocardial infarction, cardiac arrest	17 (5.8)	263 (10.0)	
All other causes of death	103 (35.4)	1157 (43.9)	
Region of birth			
South Asia	82 (28.2)	-	-
East Asia and Pacific	76 (26.1)	-	-
Latin America and Caribbean	46 (15.8)	-	-
The United States, Australia, New Zealand, and Western Europe	34 (11.7)	-	-
Middle East, North Africa, and sub-Saharan Africa	32 (11.0)	-	-
Eastern Europe and Central Asia	21 (7.2)	-	-

*Note.* Hyphen “-” represents data that are not available among long-term residents.

a Refers to areas with population less than 10 000.

b To comply with privacy regulations for minimizing the chance of identification of a study participant, numbers of participants are suppressed in the case of 5 or fewer participants (reported as ≤5 and ≤10).

c Categorized into fifths of average neighborhood income.

d Measure of the turnover in the population.

e Measure of the proportion of recent immigrants and those who self-identify as visible minority.

f Measures the size of the “dependent” population (ie, seniors and children) in relation to the “working age” population who provide social and economic support).

g Measure of inability to afford consumption goods or services.

### Outcomes

#### All potential donors

Of 2873 potential donors, 1912 families provided consent (66.6%). Families of immigrants were less likely to provide consent compared with families of long-term residents (46.4% [135 of 291] vs 68.8% [1777 of 2582]; adjusted rate ratio (RR): 0.72; 95% confidence interval [CI]: 0.63-0.81; Table 2). When examined by the region of origin, families of immigrants from different regions were less likely to consent to organ and tissue donation compared with long-term residents: South Asia (RR: 0.71; 95% CI: 0.55-0.91), East Asia and Pacific (RR: 0.68; 95% CI: 0.53-0.88) and Middle East, North Africa, and sub-Saharan Africa (RR: 0.58; 95% CI: 0.37-0.91).

**Table 2.** Rate of Familial Consent Among Immigrants and Long-term Residents (n = 2873).

Characteristic	Number consented (%)	Rate ratio (95% confidence interval)	
		Unadjusted	Adjusted^a^
World region of birth			
Long-term residents	1777 (68.8)	1.00 (Reference)	1.00 (Reference)
Immigrants (as a whole)^b^	135 (46.4)	0.67 (0.59-0.76)	0.72 (0.63-0.81)^c^
South Asia	36 (43.9)	0.64 (0.50-0.82)	0.71 (0.55-0.91)^c^
East Asia and Pacific	33 (43.4)	0.63 (0.49-0.81)	0.68 (0.53-0.88)^c^
Latin America and Caribbean	25 (54.3)	0.79 (0.61-1.03)	0.82 (0.63-1.08)
The United States, Australia, New Zealand, and Western Europe	19 (55.9)	0.81 (0.60-1.10)	0.80 (0.59-1.07)
Middle East, North Africa, and sub-Saharan Africa	12 (37.5)	0.54 (0.35-0.85)	0.58 (0.37-0.91)^c^
Eastern Europe and Central Asia	10 (47.6)	0.69 (0.44-1.08)	0.67 (0.43-1.05)

*Note.* Total number of immigrants and long-term residents who consented was 1912 (66.6%). We used complete-case analysis on 2873 patients because 53 had missing data (2.0% missing).

a Adjusted for world region of birth, sex, residence, age category, neighborhood income quintile, residential instability, material deprivation, dependency, ethnic concentration, cause of death, and academic hospital affiliation.

b Two separate analyses were conducted. One analysis adjusted for the immigrant group as a whole and the second analysis compared immigrants grouped by world region of birth with long-term residents.

c Denotes groups were less likely than long-term residents to provide familial consent.

#### Registered potential donors

Among the 2926 potential donors, 606 (20.7%) had previously registered for deceased organ and tissue donation. Among these potential donors, we found no statistically significant difference in consent rates among registered immigrants and long-term residents. Of these registered potential donors, 83.8% (31 of 37; 95% CI: 68.9%-92.7%) of immigrant families provided consent compared with 89.3% (508 of 569; 95% CI: 86.5%-91.6%) of registered long-term residents.

### Subgroup Analyses

Age, sex, and cause of death did not modify the relative association between immigrant status and familial consent (Supplemental Table 2). The relative rate of familial consent in immigrants (vs long-term residents) was lower in community hospitals compared with academic hospitals (*P* value for interaction = .045).

## Discussion

We found that families of immigrants in Ontario, Canada, were less likely to consent to deceased organ donation compared with long-term residents. However, among those who were registered for organ and tissue donation, we found no difference in the likelihood of consent.

The 2 largest ethnic groups in Ontario, Canada, are South Asians and Chinese. Similar to our previous findings that families of South Asian and Chinese individuals were less likely to provide consent,^10^ we found that families of immigrants born from the East Asia and Pacific region and South Asia were also less likely to consent to deceased organ and tissue donation even after adjustment for multiple characteristics. We also found that families of immigrants born from the Middle East, North Africa, and sub-Saharan Africa region were less likely to consent. This finding is not surprising given lower levels of support for organ donation documented within these groups.^11^

The similarity in donation rates that we observed among immigrants and long-term residents who had previously registered for organ donation supports the value of donor registries. Although our findings may have resulted from selection bias, it is also possible that the documented wishes of potential donors helped families concur with their choice to donate. In contrast, a British report found that 25% of Black and Asian families refuse to consent to organ donation even if their loved one was on the donor register compared with 10% for the rest of the population.^12^ Researchers have suggested that in situations where an individual has registered for deceased organ donation, the emphasis should be on providing families with the registration information in addition to educative and support services rather than solely focusing on obtaining familial consent.^13^ In Ontario, this has been our practice since 2009. Future research that examines differences in the reasoning behind familial refusal among immigrants compared with long-term residents may be useful to support efforts to provide educative and support services.

Our study has some limitations. First, our study was designed to measure differences in donation consent rates, and although our databases provided highly accurate information on that, we could not determine the reasons for failing to obtain familial consent for deceased organ donation. This information would be important for the design of strategies to increase consent rates and will be the subject of future work. Second, although our findings seem to support the value of organ donation registries, our analysis was limited by our small sample of immigrants and registered potential donors. Estimates from small sample sizes are imprecise, and these results should be interpreted with caution. Furthermore, while it is tempting to assume that the potential donor’s registration status helped encourage family members to provide consent, our findings may have been the result of high levels of support for organ donation throughout the families of registered potential donors. Third, our analysis of family consent among immigrants and long-term residents by world region of birth is limited by the small number of cases which yielded wide confidence intervals produced in the multivariable analysis. These estimates should be interpreted with caution given the uncertainty in the confidence intervals due to the low number of cases within the subgroup analysis. Fourth, we only had access to the potential donors’ information and not the family member who was approached for consent. It may have been possible that there are important distinguishing characteristics of families that provide (vs do not provide) consent. Fifth, many of the characteristics we examined were nonmodifiable, and this limits the number of interventions that could arise from our findings. Simpkin et al found that modifiable characteristics such as the skills of the requestor and the timing of the conversation may have a significant impact on consent rates.^14^ Sixth, an important limitation of the immigration dataset is that we classified immigrants who arrived prior to 1985 as “long-term residents” because we do not have any immigration records for immigrants prior to this date. However, as Benchimol et al mentioned, this group would have been residing in Canada for more than 10 years and their health service patterns would be more similar to nonimmigrants.^15^

Overall, our findings show that a significant number of potentially life-saving organs are going unused among all potential donors, but particularly among those of ethnic minorities. This provides an important starting point for improving the availability of organ from these subpopulations. Further research will be targeted at defining specific factors responsible for this disparity and strategies for overcoming them, including the role for expanding organ donor registration.

## Supplementary Material

Supplementary material
